# Diagnostics and clinical usability of the Montreal Cognitive Assessment (MoCA) in amyotrophic lateral sclerosis

**DOI:** 10.3389/fpsyg.2022.1012632

**Published:** 2022-09-23

**Authors:** Edoardo Nicolò Aiello, Federica Solca, Silvia Torre, Laura Carelli, Roberta Ferrucci, Alberto Priori, Federico Verde, Vincenzo Silani, Nicola Ticozzi, Barbara Poletti

**Affiliations:** ^1^Department of Neurology and Laboratory of Neuroscience, IRCCS Istituto Auxologico Italiano, Milan, Italy; ^2^PhD Program in Neuroscience, School of Medicine and Surgery, University of Milano-Bicocca, Monza, Italy; ^3^Department of Health Sciences, International Medical School, Aldo Ravelli Center for Neurotechnology and Experimental Brain Therapeutics, University of Milan, Milan, Italy; ^4^ASST Santi Paolo e Carlo, San Paolo University Hospital, Milan, Italy; ^5^IRCCS Ca’ Granda Foundation Maggiore Policlinico Hospital, Milan, Italy; ^6^Department of Pathophysiology and Transplantation, "Dino Ferrari Center", Università degli Studi di Milano, Milan, Italy

**Keywords:** Montreal Cognitive Assessment, amyotrophic lateral sclerosis, cognitive screening, diagnostics, psychometrics

## Abstract

**Background:**

The present study aimed at (1) assessing the diagnostic properties of the Montreal Cognitive Assessment (MoCA) in non-demented ALS patients and at (2) exploring the MoCA administrability according to motor-functional status.

**Materials:**

*N* = 348 patients were administered the MoCA and Edinburgh Cognitive and Behavioural ALS Screen (ECAS). Administrability rates and prevalence of defective MoCA scores were compared across King’s and Milano-Torino clinical stages. Regression models were run to test whether the non-administrability of the MoCA and a defective score on it were predicted, net of the ECAS-Total, by disease duration, ALS Functional Rating Scale-Revised (ALSFRS-R) and progression rate, computed as (48: ALSFRS-R)/disease duration. Intrinsic and post-test diagnostics were tested against a below-cut-off ECAS-total score.

**Results:**

The 79.9% of patients successfully underwent the MoCA, whose administrability rates decreased with advanced clinical stages, at variance with its defective score prevalence. The probability of the FAB not being administrable was predicted only by lower ALSFRS-R-bulbar and-upper-limb scores; no motor features, but the ECAS-Total, predicted a defective MoCA performance. The MoCA showed high accuracy (AUC = 0.82) and good intrinsic and post-test properties—being slightly more specific than sensitive.

**Discussion:**

In non-demented ALS patients, the MoCA is featured by optimal diagnostics as a screener for cognitive impairment, especially for ruling-out its occurrence, as long as patients are in the early stages of the disease and have sufficiently spared bulbar and upper-limb functions.

## Background

It is currently debated whether the Montreal Cognitive Assessment (MoCA) (Nasreddine et al., 2005), one of the most widespread, psychometrically sound and clinically usable cognitive screener (Julayanont and Nasreddine, 2017), is feasible and diagnostically adequate for use in ALS patients (Gosselt et al., 2020). Indeed, several MoCA tasks require motor−/verbal-mediated responses, which can be undermined by of upper-limb disabilities/dysarthric features.

Despite being undisputed that ALS-specific cognitive screeners, i.e., the Edinburgh Cognitive and Behavioural ALS Screen (ECAS) (Abrahams et al., 2014) and ALS Cognitive Behavioural Screen (ALS-CBS^™^) (Woolley et al., 2010)—are to be preferred over ALS-nonspecific ones in both clinical practice (Gray and Abrahams, 2022) and research (Beswick et al., 2021), it appears that the MoCA keeps being applied to this population (Gosselt et al., 2020), having also received moderate levels of recommendation (Taule et al., 2020).

However, when compared to other disease-nonspecific cognitive screeners, diagnostic and feasibility information on the MoCA in ALS patients is still currently limited (Osborne et al., 2014; Nagashima et al., 2019; Gosselt et al., 2020; Shen et al., 2020; Taule et al., 2020). Indeed, available data on its diagnostic properties have been derived from relatively small samples, as well as by not addressing ALS-specific measures as the gold-standard (Gosselt et al., 2020; Shen et al., 2020). Moreover, and most relevantly, no information is available on the interplay between the administrability of the MoCA and motor confounders in ALS.

The present study thereupon aimed at (1) assessing the diagnostic properties of the MoCA in a large, clinic-based cohort of non-demented ALS patients against an ALS-specific gold-standard measure, as well as at (2) exploring the MoCA administrability according to motor-functional status.

## Materials and methods

### Participants

The study cohort was constituted by *N* = 348 consecutive, non-demented ALS patients referred to IRCCS Istituto Auxologico Italiano between 2017 and 2021. Exclusion criteria were: (1) additional neurological/psychiatric disorders; (2) severe general-medical conditions; and (3) uncorrected hearing/vision impairments. This study was approved by the Ethics Committee of IRCCS Istituto Auxologico Italiano (I.D.: 2013_06_25); participants provided informed consent and data were treated according to current regulations.

### Materials

The Italian ECAS (Poletti et al., 2016) and MoCA (Aiello et al., 2022) were administered. The ALS Functional Rating Scale-Revised (ALSFRS-R) (Cedarbaum et al., 1999) was addressed for the assessment of disease severity, with progression rate (ΔFS) being also computed as follows: (48: ALSFRS-R)/disease duration in months (Kimura et al., 2006). Clinical stages were defined according to both King’s (Roche et al., 2012) and Milano-Torino systems (Chiò et al., 2015).

### Statistics

Chi-square tests of independence were run to compare the administrability rate and prevalence of demographically-adjusted, defective MoCA scores (Aiello et al., 2022) across King’s and Milano-Torino clinical stages.

Furthermore, logistic regression models were implemented to examine whether, net of ECAS-Total scores, motor features (i.e., disease duration, bulbar, respiratory, upper-limb and lower-limb subscores of the ALSFRS-R and ΔFS) accounted for (1) the MoCA being administrable or not and (2) a below- vs. above-cut-off MoCA score. Age, education and sex were entered as covariates into the former model only, since the MoCA cut-off is demographically-adjusted (Aiello et al., 2022). The selection of significant predictors was Bonferroni-corrected within both these models (*α*_adjusted_ = 0.05/number of target predictors, i.e., excluding covariates).

Receiver-operating characteristics (ROC) analyses were run to derive intrinsic, i.e., sensitivity (Se) and specificity (Sp), and post-test diagnostic properties, i.e., positive and negative predictive values (PPV; NPV) and likelihood ratios (LR +; LR –) – at the optimal cut-off (identified *via* Youden’s *J* statistic) against a positive state (i.e., cognitive impairment) operationalized as a defective performance on the ECAS-Total (Poletti et al., 2016). The minimum sample size was estimated, by addressing an AUC = 0.7, *α* = 0.05 and 1–*β* = 0.95 within a single-test ROC model (Kim, 2013), at *N* = 82, i.e., hypothesizing that up to 50% of patients could present with cognitive impairment (*N* = 41 cognitively-impaired vs. *N* = 41 cognitively-spared patients).

Finally, as MoCA scores distributed normally [i.e., skewness and kurtosis values ≥|1| and |3|, respectively (Goksuluk et al., 2016)], their convergence with ECAS ones was assessed by means of Bonferroni-corrected Pearson’s coefficients.

Analyses were run with R 4.1^1^ and jamovi 2.3 (the jamovi project, 2022); missing values were excluded pairwise and the α level was set at 0.05.

## Results

Table 1 shows background and clinical measures of patients being successfully administered the MoCA (278/348, i.e., 79.9% out of the initial sample).

**Table 1:** Background and clinical features of patients that underwent the MoCA.

***N***	278
Age (years)	62.8 ± 11.4 (28–88)
Sex (M/F)	64%/36%
Education (years)	11.6 ± 4.4 (5–24)
Handedness (right/left)	94.6%/5.4%
Disease duration (months)	16.7 ± 14.3 (2–120)
ALSFRS-R	
Total	39.4 ± 5.6 (22–48)
Bulbar	10.5 ± 1.9 (4–12)
Spinal – lower limbs	11.2 ± 3.9 (0–16)
Spinal – upper limbs	6.3 ± 1.7 (0–8)
Respiratory	11.3 ± 1.4 (5–12)
ΔFS	0.8 ± 0.7 (0–5.2)
KSS	
Stage 0	2%
Stage 1	36.5%
Stage 2	33.7%
Stage 3	23.3%
Stage 4	4.4%
MiToS	
Stage 0	77.5%
Stage 1	20.1%
Stage 2	2.4%
PEG	0.4%
NIV	4%
Genetics	
*C9orf72*	6.8%
*SOD1*	2.9%
*TARDBP*	3.2%
*FUS*	0.4%
MoCA	
Raw scores	23.6 ± 3.6 (11–30)
Below-cut-off scores^a^	6.1%
ECAS	
Total	100.4 ± 18.1 (39–29)
ALS-specific	74.3 ± 14.8 (22–97)
ALS-nonspecific	26.2 ± 4.9 (9–34)
Language	23.5 ± 3.9 (10–28)
Fluency	16.5 ± 5.5 (0–24)
Executive	34.3 ± 7.6 (7–47)
Memory	14.8 ± 4.5 (1–22)
Visuo-spatial	11.4 ± 1 (6–12)
ECAS-CI	0.7 ± 0.9 (0–5)

a Aiello et al. (2022).

ΔFS, progression rate; ALS, amyotrophic laterals sclerosis; ALSFRS-R, Amyotrophic Lateral Sclerosis Functional Rating Scale-Revised; ECAS, Edinburgh Cognitive and Behavioural ALS Screen; F, female; KSS=King’s staging system; M, male; MiToS, Milano-Torino staging system; MoCA, Montreal Cognitive Assessment; NIV, non-invasive ventilation; PEG, percutaneous endoscopic gastrostomy.

**Figure 1:**
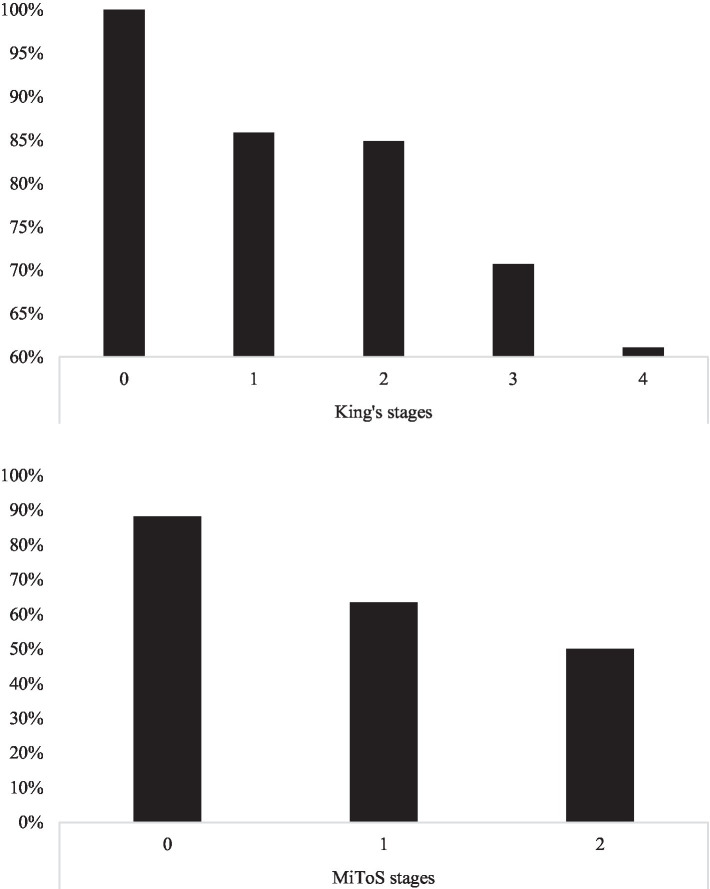
MoCA administrability rates across King’s (upper panel) and MiToS stages (lower panel). MoCA, Montreal Cognitive Assessment; MiToS, Milano–Torino staging system.

Within the whole cohort of *N* = 348 patients, the proportion of administrable MoCAs (Figure 1) decreased with advanced both King’s (*χ*^2^(4) = 13.53; *p* = 0.0029) and Milano-Torino stages (*χ*^2^(2) = 29.92; *p* < 0.001), with such a pattern not being observed as to the prevalence of defective MoCA performances (King’s: *χ*^2^(4) = 1.76; *p* = 0.780; Milano-Torino: *χ*^2^(2) = 0.46; *p* = 0.795).

The non-administrability of the MoCA was solely predicted, at *α*_adjusted_ = 0.007, by lower ALSFRS-R-bulbar (*b* = −0.29; *z* = −3.94; *p* < 0.001) and-upper-limb scores (*b* = −0.67; *z* = −5.62; *p* < 0.001), with the ECAS-Total not yielding significance (*p* = 0.458). As to patients who managed to complete the MoCA, no motor features, but only the ECAS-Total, predicted, at *α*_adjusted_ = 0.007, a below-cut-off performance on the test (*b* = −0.11; *z* = −5.24; *p* < 0.001). Supplementary Tables 1, 2 reports the results of these two models in detail. Consistently with such a statistical confirmation, the most frequent reasons for the MoCA not being administrable were (1) the presence of anarthria/severe dysarthria undermining speech intelligibility and/or (2) the loss of purposeful hand movements.

32.4% of patients successfully undergoing the MoCA were impaired on the ECAS-Total. At an optimal cut-off of 22 ≤ (*J* = 0.53; 30.9% of patients classified as impaired), MoCA raw scores proved to be highly accurate in identifying patients with a defective ECAS-Total score (AUC = 0.83; SE = 0.03; CI 95% [0.78, 0.88]), with optimal intrinsic (Se = 0.67; Sp = 0.86) and post-test properties (PPV = 0.7; NPV = 0.84; LR + = 4.82; LR – = 0.39). Similarly, when addressing age-and education-adjusted MoCA scores, optimal, although slightly lower, accuracy was reached (AUC = 0.74; SE = 0.04; CI 95% [0.67; 0.81]), with comparable intrinsic (Se = 0.67; Sp = 0.8) and post-test (PPV = 0.61; NPV = 0.83; LR + =3.3; LR– = 0.42) diagnostics, at a cut-off of < 22.381 (*J* = 0.47; 35.3% of patients classified as impaired).

At *α*_adjusted_ = 0.006, MoCA scores were significantly related to all ECAS measures (0.35 ≤ *r_s_*(278) ≤ 0.73; *p* < 0.001), with the strongest associations being found with ECAS-Total (*r* = 0.77), ECAS-ALS-specific (*r* = 0.73) and ECAS-Executive (*r* = 0.68). Supplementary Table 3 reports the full results of such correlational analyses.

## Discussion

The present study provides, for the first time, an in-depth exploration of the feasibility and diagnostics of the MoCA in non-demented ALS patients.

When compared to the ECAS, which could be administered to the whole cohort, the MoCA was feasible, pursuantly to its standardized, original procedures, in ≈ 80% of patients. However, as expected, the MoCA proved to be less frequently or not administrable to patients in the advanced disease stages, with the severity of bulbar and upper-limb involvement being inversely predictive of its feasibility. By contrast, global cognitive levels, as assessed by the ECAS, were unrelated to administrability/non-administrability of the MoCA.

Moreover, for those patients successfully undergoing the MoCA, a more advanced or severe disease did not influence its scores, which were, by contrast, predicted by their cognitive status. Taken together, such findings suggest that, as long as patients are in the early stages of the disease, and thus have sufficiently spared bulbar and upper-limb functions, the MoCA can be administered and scored without modifying its original, standardized procedures, this avoiding the entrance of unknown sources of systematic error variance in test scores (Vanderploeg, 2000). Such a stance is in line with a previous report by Osborne et al. (2014), who did not detect discrepancies in ALS patients’ MoCA scores when modifying task instructions in order to accommodate for their motor disabilities.

The diagnostic properties of the MoCA proved to be overall optimal at both intrinsic and post-test levels, although with a slight imbalance towards specificity, also indexed by a NPV higher than the PPV. Hence, it is likely that, in the ALS population, the MoCA is mostly useful for ruling-out the possibility of patients being cognitively impaired, at least, when adopting the cut-offs herewith proposed. In this respect, among the two thresholds derived within the present study, it is advisable that the one adjusted for age and education according to recent Italian norms (Aiello et al., 2022) be adopted (< 22.381).

Moreover, the significant associations detected between the MoCA and ECAS measures, and especially with ALS-specific ones, supports its construct validity in ALS patients, i.e., that this screener actually captures a set of cognitive functions that are involved in this population.

The present study is of course not fully exhaustive as to the clinical usability of the MoCA in this population. Indeed, further investigations are needed that assess its cross-sectional ability to discriminate between different neuropsychological phenotypes, as identified *via* Strong’s et al. revised classification (Strong et al., 2017).

Finally, a major *caveat* needs to be mentioned, namely that, within the present cohort, early-stage patients were overrepresented—not only in terms of disease severity and duration, but also of functional loss (Table 1). Hence, findings herewith reported on the extent to which motor-functional features affect the degree of feasibility of the MoCA, as well as its scores, should not be generalized to late-stage patients and/or to those with a markedly severe disease. Future investigations should indeed cross-sectionally address cohorts that are more representative of the full range of clinical stages, or, alternatively, explore the feasibility of the MoCA in a longitudinal fashion. With that being said, this study overall supports the notion of the MoCA being mostly feasible in ALS patients that are in the early stages of the disease.

Based on the present results, as well as on the clinical experience of the present Authors, it follows that a careful examination of bulbar and upper-limb functions needs to be carried out before determining whether the MoCA is suitable for use in this population. In this respect, an adequate strategy may be to rely on ALSFRS-R scores on items 1 (*Speech*) and 4 (*Handwriting*), although their cut-offs for determining the administrability/non-administrability of the MoCA should be derived within future studies.

In conclusion, the MoCA is a feasible and diagnostically accurate screener for global cognitive impairment in ALS patients, provided that patients are in the early stages of the disease, and thus that bulbar and upper-limb functions are sufficiently spared, and performs at its best in ruling-out the occurrence of deficits in cognition. Hence, in care settings that differ from ALS clinics (e.g., general outpatient/inpatient services, non-specialist neurology units or memory clinics), that may thus be unfamiliar with/have no expertise in adopting the ECAS (Abrahams et al., 2014) or ALS-CBS^™^ (Woolley et al., 2010), the MoCA represents a suitable and accessible alternative to screen for global cognitive impairment in this population (Simon and Goldstein, 2019). However, it is worth stressing that the MoCA does not control for verbal-motor disabilities and has not been specifically designed to cover the full range of cognitive/behavioral changes characterizing ALS patients—at variance with the ECAS, which still remains the gold-standard option for such aims in this population (Simon and Goldstein, 2019). The present study also supports the use of the MoCA in research scenarios with regard to the retrospective analysis of data collected before the availability of the ECAS or ALS-CBS^™^ (Simon and Goldstein, 2019).

## Data availability statement

The raw data supporting the conclusions of this article will be made available by the authors, without undue reservation.

## Ethics statement

The studies involving human participants were reviewed and approved by Ethics Committee of IRCCS Istituto Auxologico Italiano (I.D.: 2013_06_25). The patients/participants provided their written informed consent to participate in this study.

## Author contributions

EA: conceptualization, analyses, drafting, and revision. FS: conceptualization, data collection, drafting, and revision. ST and LC: data collection and revision. RF and AP: revision. FV, VS, NT, and BP: conceptualization, resources, drafting, and revision. All authors contributed to the article and approved the submitted version.

## Funding

This research was funded by the Italian Ministry of Health (Ricerca Corrente to IRCCS Istituto Auxologico Italiano, project 23C302). Publication fees were covered by IRCCS Istituto Auxologico Italiano.

## Conflict of interest

VS received compensation for consulting services and/or speaking activities from AveXis, Cytokinetics, Italfarmaco, Liquidweb S.r.l., and Novartis Pharma AG, receives or has received research supports from the Italian Ministry of Health, AriSLA, and E-Rare Joint Transnational Call. BP received compensation for consulting services and/or speaking activities from Liquidweb S.r.l. NT received compensation for consulting services from Amylyx Pharmaceuticals and Zambon Biotech SA.

The remaining authors declare that the research was conducted in the absence of any commercial or financial relationships that could be construed as a potential conflict of interest.

## Publisher’s note

All claims expressed in this article are solely those of the authors and do not necessarily represent those of their affiliated organizations, or those of the publisher, the editors and the reviewers. Any product that may be evaluated in this article, or claim that may be made by its manufacturer, is not guaranteed or endorsed by the publisher.

## Footnotes

^1^ https://cran.r-project.org/
