# Supplementary material for: Diagnostics and clinical usability of the Montreal Cognitive Assessment (MoCA) in amyotrophic lateral sclerosis
Source: Front Psychol. 2022 Sep 23;13:1012632. doi: 10.3389/fpsyg.2022.1012632 (PMC9540377; doi:10.3389/fpsyg.2022.1012632)
Supplement: Supplementary file 1 [file Table_1.DOCX]

| **Supplementary Table 1.** Predictors of the non-administrability of the MoCA . | | | | | | | | | |
| --- | --- | --- | --- | --- | --- | --- | --- | --- | --- |
|  |  |  |  |  |  |  |  |  |  |
| **Predictor** | | ***b*** | | ***SE*** | | ***z*** | | ***p*** | |
| Intercept |  | -6.24770 |  | 2.48386 |  | -2.5153 |  | 0.01189 |  |
| ALSFRS-R-Bulbar |  | 0.29448 |  | 0.07470 |  | 3.9423 |  | 0.00008* |  |
| Disease duration (months) |  | -0.01160 |  | 0.00836 |  | -1.3881 |  | 0.16510 |  |
| ALSFRSR-Respiratory |  | 0.22028 |  | 0.09773 |  | 2.2540 |  | 0.02420 |  |
| ALFRSR-UL |  | 0.57064 |  | 0.10146 |  | 5.6241 |  | < .00001* |  |
| ALSFRS-LL |  | -0.04971 |  | 0.05544 |  | -0.8966 |  | 0.36995 |  |
| ΔFS |  | 0.03724 |  | 0.21749 |  | 0.1712 |  | 0.86406 |  |
| ECAS-Total |  | 0.00898 |  | 0.01209 |  | 0.7425 |  | 0.45776 |  |
| Age (years) |  | -0.00286 |  | 0.01828 |  | -0.1564 |  | 0.87570 |  |
| Education (years) |  | -0.00380 |  | 0.05086 |  | -0.0748 |  | 0.94040 |  |
| Sex |  |  |  |  |  |  |  |  |  |
| F – M |  | -1.10330 |  | 0.37753 |  | -2.9224 |  | 0.00347 |  |
| **Notes.** ΔFS=progression rate; ALSFRS-R=ALS Functional Rating Scale-Revised;  ECAS=Edinburgh Cognitive and Behavioural ALS Screen; LL=lower-limb; UL=  upper-limb; *SE*=standard error. *Significant at α_adjusted_=.05/number of target predictors  (excluding covariates, *i.e.* *age, education* and *sex*)=05/7=.007. Such a model  has been run on the whole sample (*N*=348). | | | | | | | | | |
