# Supplementary material for: Diagnostics and clinical usability of the Montreal Cognitive Assessment (MoCA) in amyotrophic lateral sclerosis
Source: Front Psychol. 2022 Sep 23;13:1012632. doi: 10.3389/fpsyg.2022.1012632 (PMC9540377; doi:10.3389/fpsyg.2022.1012632)
Supplement: Supplementary file 2 [file Table_2.DOCX]

| **Supplementary Table 2.** Predictors of a below-cut-off MoCA^a^ score. | | | | | | | | | |
| --- | --- | --- | --- | --- | --- | --- | --- | --- | --- |
|  |  |  |  |  |  |  |  |  |  |
| **Predictor** | | ***b*** | | ***SE*** | | ***z*** | | ***p*** | |
| Intercept |  | 3.0656 |  | 5.7703 |  | 0.531 |  | 0.59523 |  |
| Disease duration (months) |  | -0.0490 |  | 0.0249 |  | -1.968 |  | 0.04904 |  |
| ALSFRS-R-bulbar |  | -0.1221 |  | 0.2069 |  | -0.590 |  | 0.55508 |  |
| ALSFRS-R-respiratory |  | -0.4369 |  | 0.3642 |  | -1.200 |  | 0.23030 |  |
| ALSFRS-R-UL |  | -0.3838 |  | 0.2633 |  | -1.458 |  | 0.14493 |  |
| ALSFRS-R-LL |  | -0.0320 |  | 0.1014 |  | -0.315 |  | 0.75247 |  |
| ECAS-Total |  | 0.1133 |  | 0.0216 |  | 5.239 |  | < .00001* |  |
| ΔFS |  | 0.0982 |  | 0.8260 |  | 0.119 |  | 0.90533 |  |
| **Notes.** ^a^Aiello *et al.* [13]. ΔFS=progression rate; ALSFRS-R=ALS  Functional Rating Scale-Revised;  ECAS=Edinburgh Cognitive and Behavioural ALS Screen;  LL=lower-limb; UL=upper-limb;  *SE*=standard error. *Significant at α_adjusted_=.05/number of target predictors=.05/7=.007.  Such a model has been run on those patients having been  successfully administered the MoCA (*N*=278). | | | | | | | | | |
|  | | | | | | | | | |
