# Supplementary material for: Diagnostics and clinical usability of the Montreal Cognitive Assessment (MoCA) in amyotrophic lateral sclerosis
Source: Front Psychol. 2022 Sep 23;13:1012632. doi: 10.3389/fpsyg.2022.1012632 (PMC9540377; doi:10.3389/fpsyg.2022.1012632)
Supplement: Supplementary file 3 [file Table_3.DOCX]

| **Supplementary Table 3.** Pearson’s correlations between the MoCA and ECAS score. | | | | | | | | | | | | | | | |
| --- | --- | --- | --- | --- | --- | --- | --- | --- | --- | --- | --- | --- | --- | --- | --- |
|  |  |  |  |  |  |  |  | **ECAS** |  |  |  |  |  |  |  |
|  | |  | | **Language** | | **Fluency** | | **Executive** | | **Memory** | | **Visuo-spatial** | |  | |
| **MoCA** |  | *r*(278) |  | 0.620 |  | 0.586 |  | 0.683 |  | 0.621 |  | 0.350 |  |  |  |
|  |  | *p* |  | < .00001 |  | < .00001 |  | < .00001 |  | < .00001 |  | < .00001 |  |  |  |
| **Notes.** ECAS=Edinburgh Cognitive and Behavioural ALS Screen. All the coefficients  are significant at α_adjusted_=.05/number of comparisons=.05/5=.01. | | | | | | | | | | | | | | | |
